# Intestinal intussusception in a young women: unusual cause and specific management

**DOI:** 10.1186/s12957-015-0660-0

**Published:** 2015-08-20

**Authors:** Choukri Elm’hadi, Mohamed Tarchouli, Mohamed Reda Khmamouche, Rachid Tanz, Mohammed Elfahssi, Fouad Kettani, Abdelmounaim Ait Ali, Hassan Errihani, Mohammed Ichou

**Affiliations:** Medical Oncology Department, Mohammed V Military Teaching Hospital of Rabat, zip code 10100 Rabat, Morocco; Department of Visceral Surgery, Mohammed V Military Teaching Hospital of Rabat, Rabat, Morocco; Center of Pathology United Nations, Rabat, Morocco; Medical Oncology Department, National Institute of Oncology Sidi Mohamed Ben Abdellah, Rabat, Morocco

**Keywords:** Intussusception, Ileocolic, Adenocarcinoma, Young woman, Laparoscopic surgery

## Abstract

**Background:**

Intussusception in adults is a rare cause of abdominal pain that is often associated with organic pathology. We describe a case of ileocolic intussusception revealing a cecal adenocarcinoma in a young woman successfully managed by laparoscopic-assisted surgery adhering to oncological principles.

**Case presentation:**

A 30-year-old woman with a family history of colon adenocarcinoma in a young brother presented to our emergency department with a 2-month history of intermittent colicky abdominal pain accompanied by nausea and vomiting. Physical examination showed a palpable mass in the right lower quadrant of the abdomen. Computed tomography showed a 3-layered structure giving the characteristic target-shaped appearance in the ascending colon, highly suggestive for an ileocolic intussusception associated with right colic parietal thickening and an adjacent lymphadenopathy.

Patient was planned for laparoscopic exploration and eventually definitive surgery. Intra-operatively, we found an ileocolic intussusception with thickening of the colic wall and slight proximal intestinal dilation. Multiple lymphadenopathies along the ileocecal artery were observed. Laparoscopic right hemicolectomy was performed following strict oncologic principles with “en bloc resection” and lymphadenectomy given the risk of an underlying malignancy. Considering this risk, previous reduction of the invaginated segments was not attempted and primary extracorporeal anastomosis was performed using manual sutures.

Macroscopic examination of the resected specimen revealed a tumor mass of the caecal wall .The histological analysis identified a moderately differentiated tubular adenocarcinoma invading the serosa (T3) without permeation of the lymphatic or venous capillaries .No lymphatic metastasis of 28 nodes removed was seen. Postoperative course was uneventful and patient was discharged 5 days after surgery.

Postoperative chest, abdomen, and pelvis CT scan were normal .Therefore, tumor is classified as stage II A (T3N0 M0).There was loss of MLH2 and MSH6 protein expression on immunohistochemistry findings reflecting a microsatellite instability phenotype, and the patient was followed up without adjuvant chemotherapy.

**Conclusion:**

Ileocolic intussusception rarely revealed a cancer in young adults. Laparoscopic surgery has a special interest in the diagnosis and treatment in this pathology. Oncogenetic consultation should be required in malignant lesion.

## Background

Intussusception in adults is a rare cause of abdominal pain. Unlike its pediatric counterpart, intussusception in adults is often associated with organic pathology. We describe a case of ileocolic intussusception revealing a cecal adenocarcinoma in a young woman successfully managed by laparoscopic-assisted surgery adhering to oncological principles.

## Case presentation

A 30-year-old woman with a family history of colon adenocarcinoma in a young brother presented to our emergency department with a 2-month history of intermittent colicky abdominal pain accompanied by nausea and vomiting. She denied any history of gastrointestinal bleeding, fever, or past abdominal surgery. Her appetite was good but she reported a 3-kg weight loss during the previous 3 months. Physical examination showed a palpable mass in the right lower quadrant of the abdomen, but digital rectal examination was unremarkable. This mass was firm, non-tender, and slightly painful with limited mobility, but no signs of peritoneal reaction were evident. All laboratory investigations, particularly tumor markers including carcinoembryonic antigen (CEA) and carbohydrate antigen (CA 19.9) were within the normal limits.

**Fig. 1:**
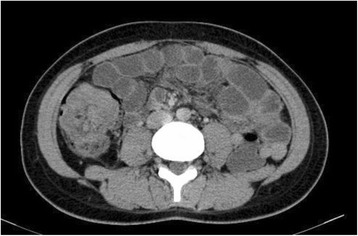
Axial CT showing intussusception at the ascending colon with wall thickening

Ultrasound revealed a prominent swelling of the intestinal wall with target signs and a hyperechoic mass over the right lower quadrant area. An abdominal computed tomography (CT) was carried out to confirm the ultrasound findings. This showed a three-layered structure giving the characteristic target-shaped appearance in the ascending colon, highly suggestive for an ileocolic intussusception (Fig. 1). Additionally, the CT showed a right colic parietal thickening with an adjacent lymphadenopathy measuring 11 × 13 mm but without signs of intestinal obstruction. Moreover, complete colonoscopy with exploration of the caecal region was paradoxically normal.

**Fig. 2:**
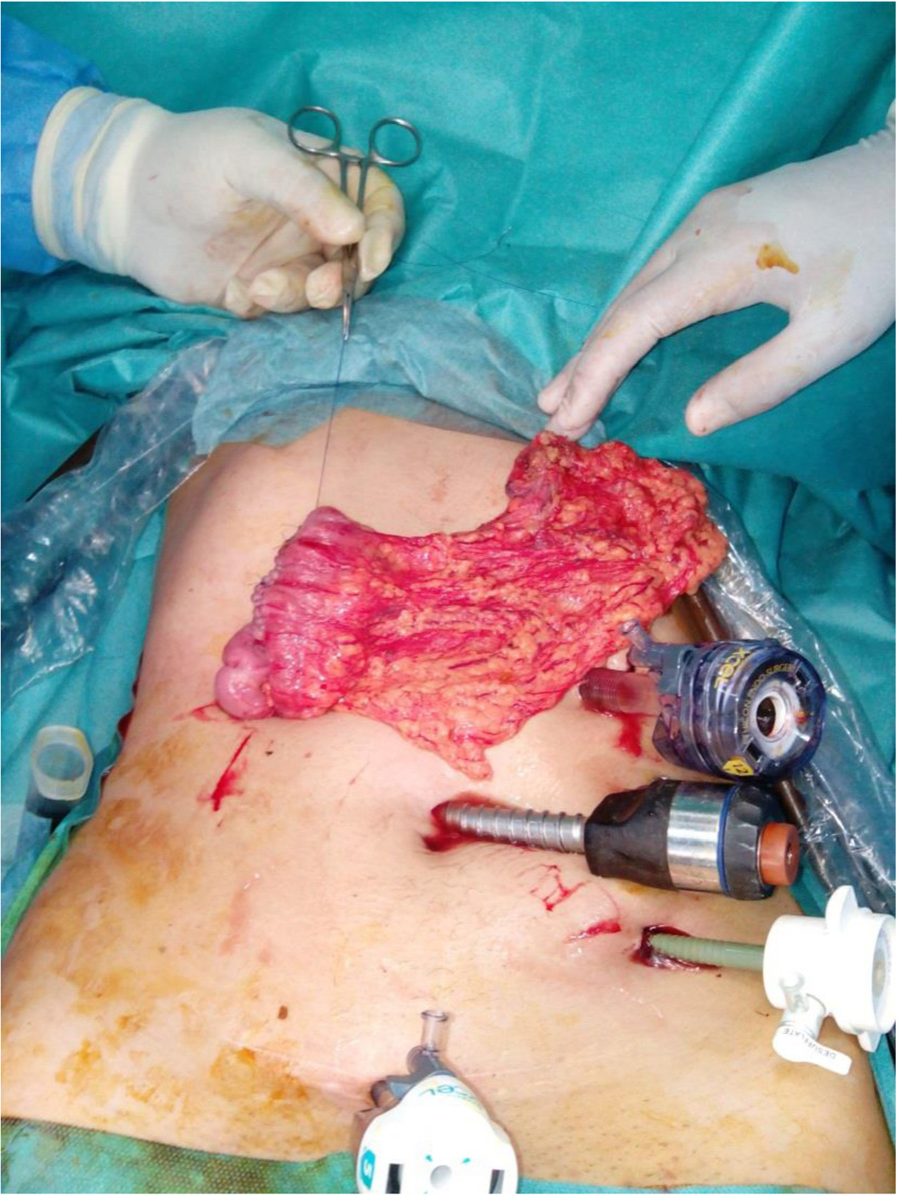
Laparoscopic right hemicolectomy

Patient was planned for laparoscopic exploration and eventually definitive surgery. Intra-operatively, we found an ileocolic intussusception with thickening of the colic wall and slight proximal intestinal dilation. In addition, multiple lymphadenopathies along the ileocecal artery were observed, but no signs of intestinal ischemia or peritoneal carcinosis were noted. Consequently, we performed a laparoscopic right hemicolectomy (Fig. 2) following strict oncologic principles with “en bloc resection” and lymphadenectomy given the risk of an underlying malignancy. Considering this risk, previous reduction of the invaginated segments was not attempted. The specimen was exteriorized through a 5-cm incision in the right upper quadrant, and primary extracorporeal anastomosis was performed using manual sutures.

**Fig. 3:**
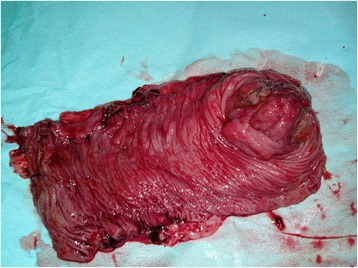
Surgical specimen after opening of the colon with appearance of tumor formation at the caecal wall

Macroscopic examination of the resected specimen revealed a tumor mass of the caecal wall measuring 4 × 5 × 4 cm and occupying more than 3-quarters of the circumference (see Fig. 3). The histological analysis identified a moderately differentiated tubular adenocarcinoma invading the serosa (T3) without permeation of the lymphatic or venous capillaries. Mucinous component was estimated at 40 %.No lymphatic metastasis of 28 nodes removed was seen and surgical margin was negative for cancer. Postoperative course was uneventful and patient was discharged 5 days after surgery.

Postoperative chest, abdomen, and pelvis CT scan were normal .Therefore, tumor is classified as stage II A (T3N0 M0). Microsatellite status and immunohistochemistry of four deoxyribonucleic acid (DNA) mismatch repair proteins, including mutL homologue 1, post meiotic segregation increased 2, mutS homologue 2, and mutS homologue 6, were checked.

**Fig. 4:**
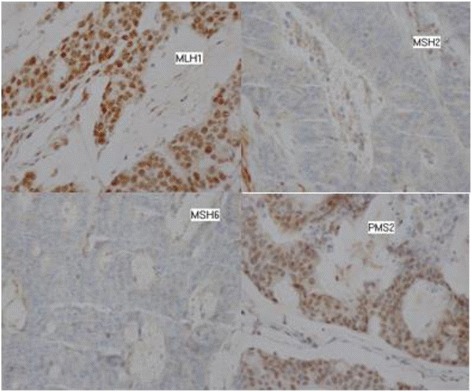
Microscopic findings and immunohistochemistry for deoxyribonucleic acid mismatch repair proteins (×200). The tumor is a moderately differentiated adenocarcinoma with immunohistochemical expression of mutL homologue 1 (*MLH1*) and postmeiotic segregation increased 2 (*PMS2*) and loss of immunohistochemical expression of mutS homologue 2 (*MSH2*) and mutS homologue 6 (*MSH6*) reflecting a microsatellite instability phenotype

There was loss of MLH2 and MSH6 protein expression on immunohistochemistry findings reflecting a microsatellite instability phenotype (Fig. 4), and the patient was followed up without adjuvant chemotherapy.

## Discussion

Intussusception is an uncommon disease in the adult population compared with that in children [1]. First described by Barbette in 1674, it is defined as the telescoping of a proximal segment of the gastrointestinal tract within the lumen of an adjacent segment [2]**.** The exact mechanism is still unknown, and it is believed that any lesion in the bowel wall or irritant within the lumen that alters normal peristaltic activity is able to initiate invagination. Unlike in children, the clinical presentation of adult intussusception is considerably various and non-specific. Most patients present with intermittent or chronic symptoms that are suggestive of incomplete intestinal obstruction. The classic pediatric triad of abdominal pain, palpable abdominal mass, and bloody stool is rarely seen [1, 3, 4].

The accuracy of computed tomography for diagnosis of adult intussusception is better than that of other examinations, including abdominal ultrasonography, barium enema study, and colonoscopy [5]. Typical features include the “target” or “sausage-shaped” mass seen at the axial and coronal view, respectively, or the appearance of bowel-within-bowel configuration with or without fat and mesenteric. The CT scan may also define the location, the nature of the mass, its relationship to surrounding tissues and additionally, it may help in disease staging in malignancy causes [6] and exploration of no specific or acute abdominal pain [7].

In this case, a preoperative diagnosis of intussusception was easily made by conventional imaging techniques including ultrasonography and CT scan despite longstanding digestive symptoms, but colonoscopy did not show the intussusception. Preoperative diagnosis of intussusception is infrequent in the adult with a rate reported to range from 32 to 70 % [8].

The optimal therapeutic management of adult intussusception remains controversial due to the differing etiology between pediatric and adult populations. A pathologic lesion is found in 90 % of adult intussusception cases [9]. Primary colon adenocarcinoma and malignant lymphoma were the two most common underlying malignant lesions [10]. Therefore, surgical exploration and bowel resection is generally recommended. However, reduction of intussusception prior to resection is deemed unsafe, considering the risk of an underlying malignancy more likely in the colic invaginations. The downsides of reduction prior to resection include the theoretical risk of intralumenal tumor seeding, reduction of externally viable bowel despite mucosal necrosis, venous embolization of malignant cells, spillage of succus through inadvertent perforation, and anastomotic complication in cases of an edematous and weakened bowel [4]. In our patient, some data suspected a malignant tumor such as: lymphadenopathy, colic parietal thickening, young age, and family history. Reducing the intestine before resection of the bowel was not performed.

Conventionally, laparotomy should be considered for the treatment. Given that laparoscopic management of pediatric intussusception has been available for some time [11] and that the long-term safety of laparoscopic resection for colorectal cancer is well established, laparoscopic-assisted surgery currently appears to be an attractive alternative to laparotomy in selected patients. This procedure entails mobilization and delivering the specimen with an extracorporeal anastomosis in most patients. This requires utmost care and an experienced laparoscopic surgeon.

We herein describe a laparoscopic treatment for adult colonic intussusception caused by cecum carcinoma. In the present case, laparoscopic right hemicolectomy was successfully performed. The experience gained in the present case suggests that laparoscopy may be a diagnostic or therapeutic tool for selected cases of young adult intussusception.

The histological examination showed a cecal adenocarcinoma stage II A according to the TNM classification, for which the indication of adjuvant chemotherapy is subject of controversy. Our patient presented more favorable prognostic factors such that: tumor was pT3, moderately differentiated and without angiolymphatic, or perineural invasion. Node dissection was optimal, and surgical margins were negative. Microsatellite was instable, and patient can be spared adjuvant chemotherapy [12].

Finally, many items are in favor of a lynch syndrome according to BETHESDA criteria; colon cancer was diagnosed before the age of 50 years, with one first-degree relative with an HNPCC-related tumor diagnosed under age 50 years. The abundant mucus is presented, and microsatellite was an instable phenotype with loss of expression of MSH2 and MSH 6 which often reflects a germline mutation and therefore an HNPCC. Lynch syndrome is suspected, and cancer genetics consultation is required [13].

## Conclusions

Ileocolic intussusception rarely revealed a cancer in young adults. It can detect a genetic predisposition. Laparoscopic surgery has a special interest in the diagnosis and treatment in this pathology. Oncogenetic consultation should be required in a malignant lesion.

## Consent

Written informed consent was obtained from the patient for publication of this Case report and any accompanying images. A copy of the written consent is available for review by the Editor-in-Chief of this journal.

## Abbreviations

CA 19.9: carbohydrate antigen
CEA: carcinoembryonic antigen
CT: computed tomography
DNA: deoxyribonucleic acid
HNPCC: Hereditary non polyposis colorectal cancer
MLH: mutL homologue
MSH: mutS homologue
TNM: tumor nodes metastasis
